# Improving the assessment of quality of life in the clinical care of myeloma patients: the development and validation of the Myeloma Patient Outcome Scale (MyPOS)

**DOI:** 10.1186/s12885-015-1261-6

**Published:** 2015-04-14

**Authors:** Thomas R Osborne, Christina Ramsenthaler, Stephen A Schey, Richard J Siegert, Polly M Edmonds, Irene J Higginson

**Affiliations:** 1 King’s College London Department of Palliative Care, Policy and Rehabilitation, Cicely Saunders Institute, London, UK; 2 Department of Haematological Medicine, King’s College Hospital and King’s College London, London, UK; 3 School of Public Health and Psychosocial Studies and School of Rehabilitation and Occupation Studies, Auckland University of Technology, Auckland, New Zealand; 4 Department of Palliative Care, King’s College Hospital, London, UK

**Keywords:** Cancer, Oncology, Haematology, Multiple myeloma, Quality of life, Outcome assessment, Psychometrics

## Abstract

**Background:**

Multiple myeloma is an incurable cancer with a rising incidence globally. Less toxic treatments are increasingly available, so patients are living longer and treatment decisions are increasingly guided by QOL concerns. There is no QOL assessment tool designed specifically for use in the clinical care of people with myeloma. This study aimed to develop and test the psychometric properties of a new myeloma-specific QOL questionnaire designed specifically for use in the clinical setting – the MyPOS.

**Methods:**

The MyPOS was developed using findings from a previously reported literature review and qualitative study. The prototype MyPOS was pretested using cognitive interviews in a purposive sample of myeloma patients and refined prior to field testing. The psychometric properties of the MyPOS were evaluated in a multi-centre, cross sectional survey of myeloma patients recruited from 14 hospital trusts across England.

**Results:**

The prototype MyPOS contained 33 structured and open questions. These were refined using cognitive interviews with 12 patients, and the final MyPOS contained 30 items taken forward for field-testing. The cross-sectional survey recruited 380 patients for the MyPOS validation. Mean time to complete was 7 minutes 19 seconds with 0.58% missing MyPOS items overall. Internal consistency was high (α = 0.89). Factor analysis confirmed three subscales: Symptoms & Function; Emotional Response and Healthcare Support. MyPOS total scores were higher (worse QOL) in those with active disease compared to those in the stable or plateau phase (F = 11.89, p < 0.001) and were worse in those currently receiving chemotherapy (t = 3.42, p = 0.001). Scores in the Symptoms & Function subscale were higher (worse QOL) in those with worse ECOG performance status (F = 31.33, p < 0.001). Good convergent and discriminant validity were demonstrated.

**Conclusions:**

The MyPOS is the first myeloma-specific QOL questionnaire designed specifically for use in the clinical setting. The MyPOS is based on qualitative enquiry and the issues most important to patients. It is a brief, comprehensive and acceptable tool that is reliable and valid on psychometric testing. The MyPOS can now be used to support clinical decision making in the routine care of myeloma patients.

**Electronic supplementary material:**

The online version of this article (doi:10.1186/s12885-015-1261-6) contains supplementary material, which is available to authorized users.

## Background

Multiple myeloma is a malignant proliferation of plasma cells with over 114,000 new cases globally in 2012 [1] and an increasing incidence worldwide [2]. Myeloma causes destruction of the bones, bone marrow failure and renal failure, leading to impairments in physical, psychological and social domains of quality of life (QOL) [3-5]. Myeloma remains incurable, but median survival has increased from 24–32 months in 1992 to 68 months in 2005 due to increased availability of less toxic drugs [6]. Patients are now living longer with the complications of their illness, so clinical decision-making is increasingly driven by QOL concerns. It has been recommended that QOL assessment should form part of the routine care of myeloma patients [3,4,7].

A systematic literature review identified 13 QOL tools developed or validated for use in people with myeloma [8]. The review identified no tool designed specifically for clinical use, and only one disease-specific questionnaire: the European Organisation for Research and Treatment of Cancer core cancer questionnaire (EORTC-QLQ-C30) with its myeloma-specific module (MY20) [9-11]. Subsequent to the literature review, one further myeloma-specific questionnaire has been described: the Functional Assessment of Cancer Therapy – Multiple Myeloma (FACT-MM) [12]. The literature review revealed a paucity of studies to fully characterise the meaning of QOL from the perspective of myeloma patients, and concluded that the best existing QOL questionnaires may not capture all the issues important to QOL [8]. Therefore, a detailed qualitative study has subsequently explored the meaning of QOL from the perspective of people with myeloma, obtained views on a range of existing QOL questionnaires and reported a theoretical model of QOL in myeloma [13]. This model further highlighted that existing questionnaires do not capture all the issues, for example by not including items on health service factors and sexual function that are important to patients. The model suggested that the presence or absence of physical symptoms *per se* was not the most important determinant of QOL, but rather the *impact* of symptoms on other domains such as activities, participation, and emotional wellbeing. Most existing QOL questionnaires ask only about symptom *status* [8] and so may not capture all that is important to QOL.

These findings were used to develop the Myeloma Patient Outcome Scale (MyPOS) – a new QOL assessment tool designed for use within the clinical care of myeloma patients. The aim of the present article is to describe the development, pretesting and psychometric evaluation of the MyPOS questionnaire.

## Methods

### Study design

The development of the MyPOS was overseen by the MyPOS steering group, comprising experts from the fields of haematology, palliative care, psychology and psychometrics. Initially the steering group oversaw the development of a prototype MyPOS. The prototype questionnaire was pretested using cognitive interviews in a purposive sample of myeloma patients with subsequent refinements prior to field testing. Finally, the psychometric properties of the MyPOS were evaluated in a multi-centre, cross sectional survey of myeloma patients recruited from 14 hospital trusts across England. This study forms part of a wider programme of work to improve the assessment of QOL in the clinical care of myeloma patients.

### Prototype MyPOS development

It was considered preferable to modify an existing questionnaire rather than design a completely new tool, to take advantage of existing development work and relevant items that had been field-tested and used in clinical practice. The literature review had identified that the EORTC-QLQ-C30 and MY20 had undergone the most extensive psychometric validation in myeloma patients, but that these tools were designed for use in research and are predominantly health status questionnaires that may not be well suited to clinical use [8]. To align better with the findings of the earlier qualitative study [13], the steering group sought to adapt a tool that required respondents to consider the *impact* of physical symptoms on wider experience, rather than just symptom *status*. There was no suitable candidate identified in the systematic review, so the steering group chose to adapt an alternative tool – the Palliative Care Outcome Scale (POS) with its accompanying symptoms scale (POS-S) [14]. The POS was chosen because it was designed as a clinical tool and is suitable for use in any chronic illness, with many issues and themes applicable to myeloma. Importantly, the response options used in the POS-S ask the respondent to consider the *impact* of physical symptoms on ‘activities and concentration’, rather than just symptom status.

Content validity of the MyPOS was ensured by basing the items on the issues most important to patients. The earlier qualitative study and theoretical model identified 80 issues important to QOL [13]. These were refined into a 33-item prototype MyPOS using a combination of structured and open questions. Physical symptoms were only included as structured items if raised by at least 2 participants in the qualitative interviews. If raised by only one participant, symptoms were not included as structured items, with open questions used to capture any less commonly occurring symptoms.

The layout and length of the prototype MyPOS were based on the preferences of myeloma patients and clinical staff, also identified in the earlier qualitative work: the target length was no more than 2 A4 pages; items with identical response options were grouped together to reduce the amount of reading for respondents and allow information on completed questionnaires to be more easily assimilated by clinical staff; and the questionnaire contained a mixture of structured and open questions to give respondents an individualised voice to focus the goals of care on what is most important to patients [13].

### Cognitive interviews

#### Participants and setting

Participants for the cognitive interviews were recruited from inpatient and outpatient settings at King’s College Hospital NHS Foundation Trust, which provides tertiary haemato-oncology services to London and south-east England and contains the largest bone marrow transplant centre in Europe. Inclusion criteria were those 18 years or older; a confirmed histological diagnosis of multiple myeloma; being aware of the diagnosis; and capacity to give written informed consent. Exclusion criteria were those too unwell, symptomatic or distressed to participate (as judged by the clinical team); severe neutropenia where contact with researcher may pose a risk; and unable to understand written and spoken English.

Participants were purposively sampled by gender, age (<65 or ≥65), Eastern Co-operative Oncology Group (ECOG) performance status (0–2 or 3–4) and disease phase (newly diagnosed, plateau, or relapsed [15]). Purposive sampling was used to achieve maximum variation across key characteristics thought to potentially influence participants’ views and ability to understand the MyPOS items. The interviews were conducted in rounds of 6, after which recruitment was paused, interviews analysed and MyPOS refined prior to the next round 6 interviews. The number of interviews was not fixed at the start, but recruitment was continued until no further cognitive testing was required. There is no accepted best practice for the number of interviews required [16], but it has been reported that 7–10 interviews are generally sufficient [17].

#### Procedure

Cognitive interviews were used to explore the cognitive processes employed by respondents as they completed the prototype MyPOS. The interviews included a combination of think-aloud and verbal-probing techniques to evaluate each item [16,18]. Participants completed the prototype MyPOS and were asked to report what they were thinking as they answered each item (think-aloud). In practice, many participants required some direct questioning from the interviewer to clarify the issues of interest (verbal-probing). The interviewer probes were standardised using an interview topic guide based on the four-stage model of question response proposed by Tourangeau: comprehension, retrieval, judgement and response [19]. Specific probes were developed for each of these stages, adapted from examples proposed elsewhere [20] with additional questions to assess acceptability of items. The cognitive probes used are shown in Additional file 1: Table S1.

Interviews took place in a private room with only the interviewer and participant present to reduce bias in the participants’ responses. Interviews were audio recorded and conducted by a single researcher (TRO) who has a background as a medical doctor in both haemato-oncology and palliative medicine.

#### Analysis

Interviews were analysed directly from audio recordings without transcription [21]. Data were extracted into tables constructed with participant numbers across the top and questionnaire items down the left hand column. For each item, the table contained a row for each stage of question response (comprehension, retrieval, judgement, response), with additional rows for acceptability and other comments. Once the table had been populated analysis could take place ‘item-by-item’ where all views about each item could be considered in aggregate. Data were extracted and analysed for all interviews by TRO. For each round of 6 interviews a second researcher (CR) double-extracted a single interview (randomly selected) to check for consistency. Discrepancies were resolved by consensus. After the completion of each round of 6 interviews, any necessary refinements were made to the MyPOS and then 6 further interviews carried out. This was repeated until no further modifications were needed and the MyPOS then taken forward for psychometric testing.

### Cross sectional survey

#### Participants and setting

Participants for the cross sectional survey were recruited from outpatient clinics and inpatient wards at 14 hospital trusts across England, with King’s College Hospital NHS Foundation Trust acting as lead site. The collaborating trusts included a mixture of tertiary and district general hospitals to ensure the MyPOS was tested in a range of settings (see Acknowledgments full list of collaborators). Inclusion and exclusion criteria were the same as those used for the cognitive interviews. The survey recruited consecutive patients whereby all available myeloma patients were screened for eligibility at every outpatient clinic or ward where recruitment was active. All eligible patients were asked if they would participate in the study. All non-participants (those who were ineligible and those who declined) were asked for consent to record limited demographic and treatment details in order to compare these against the study sample. The recruitment target for the cross sectional survey was estimated based on the number of MyPOS items. Published estimates of the required participant to item ratio for psychometric analysis vary from 10:1 [22] to 2:1 [23]. The final MyPOS contained 27 items for psychometric evaluation so a sample of 350 was sought as a conservative estimate with an allowance for missing data.

#### Procedure

Demographic and clinical characteristics were recorded by research staff at the time of consent. Participants were given the option of completing the questionnaire booklet at the time of consent or taking it home and returning by post. The questionnaire booklet contained the MyPOS alongside the EORTC-QLQ-C30 and MY20 for validation purposes. These questionnaires were chosen as they have undergone the most extensive psychometric validation in myeloma patients [8]. The EORTC-QLQ-C30 has 30 items broken into 5 function scales (Physical, Role, Cognitive, Emotional, and Social Function); 3 symptom scales (Fatigue, Pain, and Nausea/Vomiting); a Global Health Status/QOL scale; and 6 single items (Constipation, Diarrhoea, Insomnia, Dyspnoea, Appetite Loss, and Financial Difficulties). Higher scores for function and global scales represent better QOL, whereas higher scores for symptom scales and single items represent worse QOL. The MY20 has 20 items broken into 3 subscales (Disease Symptoms, Side Effects of Treatment, and Future Perspective), and a single item for Body Image. The Future Perspective scale includes worry about death, worry about health in the future, and thinking about the illness. Higher scores for Disease Symptoms and Side Effects of Treatment scales represent worse QOL, whereas higher scores for Future Perspective and Body Image represent better QOL.

#### Analysis

All demographic and questionnaire data were double entered from paper booklets into electronic databases by two separate research staff. The two resulting databases were compared and discrepancies resolved by referring back to source data.

*Descriptive statistics* were used to describe the sample and the range and distribution of scores for individual MyPOS items and identified subscales. The differences between participants and non-participants were explored in terms of gender, age (dichotomised to <65 and ≥65), phase of disease (newly diagnosed/stable or plateau/relapsed or progressive), and treatment status (on/off treatment).

*Acceptability* was assessed by computing the proportion of missing responses per item, per subscale and overall, and by measuring time taken to complete the MyPOS in a subsample of 70 participants recruited at the lead site.

*Structural validity* was established using exploratory factor analysis to identify underlying subscales. The principal component model with Promax rotation was used. Choice of oblique (Promax) rotation was based on the theoretical model of QOL in myeloma which indicated that any potential factors might be correlated, due to the inter-relatedness of emotional issues, symptoms, activities, participation and support factors [13]. Factorability of the matrix was assessed by item intercorrelations (cut-off: <.30 [24]), Barlett’s test of sphericity [25] and the Kaiser-Meyer-Olkin test of sample adequacy [26,27]. The number of factors was determined by using three methods: The Kaiser criterion (eigenvalues > 1); scree plot [28]; and Velicer’s Minimum Average Partial (MAP) test [29]. The latter method has been suggested as more robust than the Kaiser criterion or scree plot, preventing over- or underestimation of the number of factors [30]. The resulting pattern matrix was checked for parsimony of factors. Items loading on more than one factor or with small loadings were discussed in the steering group and kept in the model if they were felt to be important on clinical grounds.

*Reliability* was estimated using Cronbach’s α for MyPOS total scores and any identified subscales. An α-coefficient in the range 0.7-0.9 is considered desirable to indicate good internal consistency without redundancy of items [31-33].

*Construct validity* was tested using the known-group comparison method [31]. It was hypothesised that (i) MyPOS scores would be higher (worse QOL) in those with active disease (newly diagnosed or relapsed) compared to those in the stable or plateau phase; (ii) MyPOS scores would be higher (worse QOL) in those currently receiving chemotherapy compared to those who were off treatment; and (iii) MyPOS scores would be higher (worse QOL) in those with worse ECOG performance status. Parametric tests were used in each case, but all comparisons were also run using non-parametric tests to account for non-normally distributed data (ANOVA and Kruskal-Wallis H for phase of disease and ECOG performance status; t-test and Mann–Whitney U for comparison of treatment status).

*Convergent and divergent validity* were tested by correlating subscales from the MyPOS with those from the EORTC-QLQ-C30 and MY20. Scores from the EORTC tools were transformed to a 0–100 scale [34] and correlated with MyPOS scores using Pearson product–moment correlation coefficients with associated p values. A strong correlation was considered to be r > 0.70 and moderate correlation r > 0.50. The minimum relevant correlation was considered to be *r* > 0.50. It was hypothesised that (i) MyPOS total score would have moderate or strong *negative* correlation with EORTC-QLQ-C30 Global Health Status/QOL scale (high MyPOS scores represent worse QOL whereas high EORTC scores represent better QOL); (ii) MyPOS symptom and function items would have moderate or strong *negative* correlation with EORTC-QLQ-C30 Physical Function, Role Function, Cognitive Function and Social Function scales, and moderate or strong *positive* correlation with MY20 Disease Symptoms and Side Effects of Treatment scales; (iii) MyPOS emotional wellbeing items would have moderate or strong *negative* correlation with the EORTC-QLQ-C30 Emotional Function and MY20 Future Perspectives scales, and (iv) MyPOS healthcare items would not correlate strongly with any EORTC scale since these issues are not captured in the EORTC questionnaires.

Statistical analyses were carried out using the Statistical Package for the Social Sciences (IBM SPSS Statistics for Windows, Version 21.0, Armonk, NY: IBM Corp). A p-value of <0.05 was considered statistically significant for all analyses. Missing data were excluded pairwise for all analyses.

### Ethical issues

Research Ethics Committee approval was granted by the South East London REC-3 (ref 10/H0808/133). All patients were screened by a member of their clinical team before being approached about participation in the study. All participants gave written consent to take part. Participation was voluntary and interviews or questionnaire completion took place at a time and place convenient to participants (hospital, home or other location requested by them). Completed questionnaires were screened for clinically important issues and where necessary the participant’s consent was sought to feed such issues back to the clinical team.

## Results

### Prototype MyPOS development

The prototype MyPOS contained 33 items including 31 structured and 2 open questions. These were composed of 10 existing POS items (those relevant to QOL in myeloma) and 23 newly written items. All response options were taken from the POS and POS-S, although the recall period was amended from 3 days to 1 week to match the preferences of myeloma patients and staff identified in the previously reported qualitative interviews [13]. Prototype MyPOS items are shown in Additional file 1: Table S2.

### Cognitive interviews

Fifteen eligible patients were approached about the cognitive interviews and 12 agreed to participate. Reasons for declining were *feeling too unwell* (2) or *no reason given* (1). Participants were recruited over a 3 month period, with interviews taking place in mixture of settings including hospital outpatients (3), hospital inpatient (3) and the participants’ own homes (6). A balance of participants was achieved across key demographic and clinical characteristics as shown in Additional file 1: Table S3.

After the first round of 6 interviews some items and response options were reworded. The reworded items were re-tested in a second round of 6 interviews. All reworded items tested well in the second round of interviews, with no further refinement needed. The only changes made after the second round were the removal of 2 items considered to be redundant and changes to layout. Further cognitive testing of these changes was not deemed necessary and so recruitment was stopped after 12 interviews. A summary of changes to the prototype MyPOS following the cognitive interviews is shown in Additional file 1: Table S4.

The MyPOS taken forward for psychometric testing contained 30 items, including 2 open questions and 1 question asking if any help was received when completing the questionnaire. 27 items were therefore included in the psychometric analysis, each scored on a 5-point scale from 0 (better QOL) to 4 (worse QOL). The item about sex-life contained an additional “*prefer not to answer”* response, which was treated as missing data for the purposes of psychometric analysis. The complete MyPOS taken forward for psychometric testing is shown in Additional file 1: Table S5.

### Cross sectional survey

517 patients with multiple myeloma were screened by the clinical teams. 465 patients were eligible against the inclusion and exclusion criteria and were approached about the study. 401 patients consented to participate, and completed questionnaires were received from 380 participants. Median age of participants was 69 years (range 38–91). There were more men (60.8%) than women (39.2%), and 181 participants (47.6%) were currently receiving treatment for their myeloma (Table 1).

**Table 1:** **Sample characteristics for cross sectional survey (n = 380)**

***Setting of questionnaire completion***	
Hospital outpatient	266 (70%)
Hospital inpatient	18 (4.7%)
Participant’s home	93 (24.5%)
Other/not known	3 (0.8%)
***Gender***	
Male	231 (60.8%)
Female	149 (39.2%)
***Age***	
Median (range)	69 (38–91)
<65	113 (29.7%)
≥65	267 (70.3%)
***Marital status***	
Single	33 (8.7%)
Married / partnered	275 (72.4%)
Divorcer / separated	26 (6.8%)
Widowed	45 (11.8%)
Not known	1 (0.3%)
***Ethnicity***	
White British	326 (85.8%)
White Other	27 (7.1%)
Black	12 (3.2%)
Asian	10 (2.6%)
Other	5 (1.3%)
***Religion***	
Atheist	34 (8.9%)
Christian	305 (80.3%)
Muslim	7 (1.8%)
Other	21 (5.5%)
Not known	13 (3.4%)
***Highest educational level***	
Did not finish school	27 (7.1%)
Secondary school graduate	183 (48.2%)
College / technical qualification	88 (23.2%)
University first degree	37 (9.7%)
University higher degree	21 (5.5%)
Not known	24 (6.3%)
***Occupation status***	
Working or student	49 (12.9%)
Not working	47 (12.4%)
Retired	283 (74.5%)
Not known	1 (0.3%)
***ECOG performance status***	
0	133 (35.0%)
1	146 (38.4%)
2	64 (16.8%)
3	36 (9.5%)
4	1 (0.3%)
***Treatment status***	
Off treatment	199 (52.4%)
On treatment	181 (47.6%)
- High dose treatment with stem cell support	5 (1.3%)
- Chemotherapy / targeted therapy alone	141 (37.1%)
- Maintenance treatment	35 (9.2%)
***Disease phase***	
Newly diagnosed	79 (20.8%)
Stable / plateau phase	174 (45.8%)
Relapsed / progressive	127 (33.4%)
***Immunoglobulin type***	
IgG	227 (59.7%)
IgA	79 (20.8%)
IgM	3 (0.8%)
Light chain	65 (17.1%)
Non-secretory	4 (1.1%)
Not known	2 (0.5%)
***ISS Stage at diagnosis***	
I	105 (27.6%)
II	83 (21.8%)
III	81 (21.3%)
Not known	111 (29.2%)
***Months since diagnosis***	
0-12	101 (26.6%)
13-24	63 (16.6%)
25-36	31 (8.2%)
37-48	36 (9.5%)
Over 48	127 (33.4%)
Not known	22 (5.8%)

There were 137 non-participants of which 52 were ineligible against the inclusion and exclusion criteria, 64 declined to participate and 21 consented but the completed questionnaire was not received. Reasons for ineligibility were being too unwell, symptomatic or distressed to participate (17); unable to understand written and spoken English (13); lack of capacity to give written informed consent (7); no histological confirmation of diagnosis (6); not yet told the diagnosis (1); and ‘other’ (8). Reasons for declining were feeling too unwell to participate (14); feeling short of time (7); privacy concerns (4); participating in other studies (3); other (8); and not known (28). Comparing the 380 survey participants to the 137 non-participants showed that the groups had similar distributions in terms of gender, age, phase of disease and treatment status. However, non-participant demographics and clinical characteristics were incomplete since such data could not be collected without consent due to ethical and data protection considerations (see Additional file 1: Table S6).

#### Acceptability

The mean time to complete the MyPOS (n = 70) was 7 minutes and 19 seconds (SD: 3 minutes 43 seconds). Median time to complete was 7 minutes (range 1 minute 55 seconds – 20 minutes). 279 participants (73.4%) completed the MyPOS without assistance; 66 (17.4%) with help from a friend or relative; and 35 (9.2%) with help from a member of staff. Ten out of 27 items had 100% response rate with no missing data. Seventeen of 27 items had missing data points with the highest rate of missing data for item about sex (“Over the past week, have you been worrying about your sex life?”) with 32 (8.4%) missing responses including 31 participants who responded *“*prefer not to answer*”* and 1 who left the item blank. The percentage of missing item-responses overall was 0.58%.

#### Structural validity and identification of subscales

Principal component analysis was carried out to explore the underlying component (subscale) structure of the MyPOS. Applying the Kaiser criterion (eigenvalues > 1) suggested a 7-component solution. The scree plot was difficult to interpret due to a strong first component, and suggested either a 2, 3 or 7 component solution. Velicer’s MAP test suggested a 3-component solution. These solutions were all tested. As the 3-component solution supports the factors derived from the theoretical model of QOL in myeloma, this was forced in the SPSS programme. Table 2 shows the pattern matrix with a forced three-component solution and after Promax rotation with missing data excluded pairwise. The first component (Symptoms & Function) comprised 14 items, which included an item about diarrhoea that had a factor loading of below 0.30. This indicates that responses to the Diarrhoea item did not correlate closely with other items in the Symptoms & Functions subscale. The Diarrhoea item also failed to load on any other component to >0.30. However, diarrhoea was considered to be an important clinical symptom, so the item was retained in the Symptoms & Function subscale on clinical grounds. The second component (Emotional Response) comprised 8 items about depression, anxiety, specific worries and ability to cope with illness and treatment. The third component (Healthcare Support) comprised 5 items about the accessibility and standard of healthcare and information received about the illness and treatment. Overall, the three components explained 41.2% of the variance. The first component (Symptoms & Function) explained 27.2%; the second component (Emotional Response) explained 8.0%; and the third component (Healthcare Support) 6.1%. All items loaded onto the subscale predicted by the model. The principal component analysis was run again with missing data excluded listwise and yielded the same factor structure.

**Table 2:** **MyPOS principal component pattern matrix with Promax rotation, forced extraction of three components, missing data excluded pairwise (n = 380)**

Abbreviated item wordings^a^	Components			Corrected item-total correlation
	(1) Symptoms & function	(2) Emotional response	(3) Healthcare support	
Pain	***.62***	.08	-.01	.58
Fatigue or lack of energy	***.80***	.01	-.17	.59
Shortness of breath	***.59***	.01	-.17	.40
Diarrhoea	*.25*	.15	-.14	.25
Constipation	***.45***	-.14	.15	.34
Nausea	***.45***	.11	.05	.47
Vomiting	***.39***	-.07	.04	.28
Mouth problems	***.30***	.27	-.19	.36
Poor mobility	***.88***	-.15	-.01	.62
Tingling in the hands and/or feet	***.47***	-.18	.06	.28
Difficulty remembering things	***.40***	.20	-.20	.39
Usual activities without help from others	***.74***	.04	.04	.66
Hobbies and leisure activities	***.74***	.00	.07	.66
Quality time with family and friends	***.58***	-.03	.18	.54
ᅟ				
Worry about sex life	-.30	***.74***	-.21	.26
Feeling depressed	.17	***.53***	.06	.59
Anxious or worried about illness or treatment	.13	***.63***	.16	.65
Worry about infections	.11	***.55***	-.04	.46
Worry about physical appearance	-.06	***.64***	.06	.44
Worry about financial situation	-.12	***.73***	.05	.45
Worry that illness will get worse	.05	***.67***	.15	.60
Able to cope with your illness and treatment	**.33**	***.39***	.14	.63
ᅟ				
Doctors or nurses – contact if needed	.01	.04	***.66***	.36
Doctors and nurses – knowledge and skill	-.06	-.04	***.86***	.33
Doctors and nurses – care and respect	-.14	-.00	***.81***	.28
Enough information about illness and treatment	-.05	.02	***.60***	.32
Enough information about the future	.22	-.02	***.49***	.43
ᅟ				
Eigenvalue	7.34	2.15	1.64	-
Percentage variance explained	27.2	8.0	6.1	-
Cumulative percentage variance explained	27.2	35.1	41.2	-

^a^Full item wordings are shown Additional file 1: Table S5.

Figures in bold indicate loadings ≥ .30.

Figures in italics indicate the component/subscale to which each item is assigned.

#### Item descriptive statistics

Participants used the full range of response options on the 5-point scale for all except three items. For the item about Vomiting, participants used the lower four responses only with none using the most severe ‘Overwhelming’ option. For item about knowledge and skill of doctors and nurses, participants used the lower four responses only, with none using the worst option ‘Not at all’. For the item about care and respect of doctors and nurses, only the lower 3 options were used with none using the worst two options ‘Occasionally’ or ‘Not at all’. Most items showed positive skew. Descriptive statistics by MyPOS subscale are shown in Table 3.

**Table 3:** **MyPOS subscale descriptive statistics**

Subscale	Number of items	Possible range	Observed range	Mean	SD	Skew	α^a^
MyPOS Symptoms & Function	14	0-56	0-37	13.46	8.25	0.37	0.84
MyPOS Emotional Response	8	0-32	0- 23	6.12	5.30	0.96	0.82
MyPOS Healthcare Support	5	0-20	0-12	1.76	2.43	1.60	0.64

^a^Cronbach’s alpha coefficient of internal reliability.

#### Reliability

The MyPOS total score showed good internal consistency with Cronbach’s α = 0.89, which was within the desired range of 0.7-0.9. Cronbach’s α was also in the desired range for all MyPOS subscales except the Healthcare Support scale for which α = 0.64 (Table 3).

#### Construct validity (known-group comparisons)

**Figure 1:**
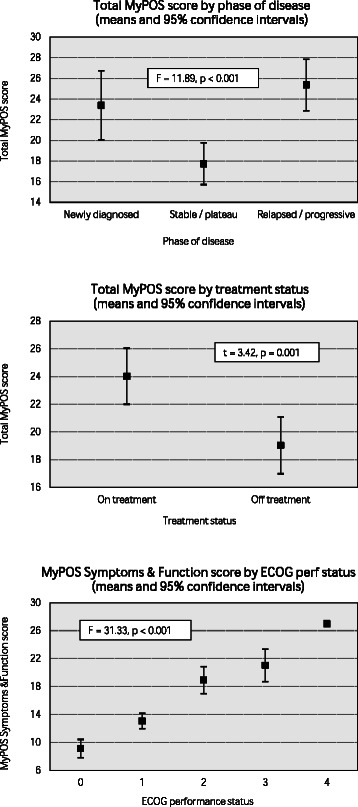
Known group comparisons showing MyPOS total score by phase of disease; MyPOS total score by treatment status; and MyPOS symptoms & function score by ECOG performance status.

All tested hypotheses were confirmed: parametric testing showed that MyPOS total scores were higher (worse QOL) in those with newly diagnosed and relapsed or progressive disease compared to those with stable disease (F = 11.89, p < 0.001); MyPOS total scores were higher (worse QOL) in those currently receiving chemotherapy compared to those not on treatment (t = 3.42, p = 0.001); MyPOS Symptoms & Function subscale scores were higher (worse QOL) in those with worse ECOG performance status (F = 31.33, p < 0.001). Figure 1 shows theses result using parametric testing. Due to skewed data these comparisons were also run using equivalent non-parametric tests with highly similar results.

#### Convergent and divergent validity

All tested hypotheses were confirmed: MyPOS total scores correlated negatively with EORTC-QLQ-C30 Global Health Status/QOL (r = −0.70, p < 0.001); MyPOS Symptoms & Function scores correlated negatively with EORTC-QLQ-C30 Physical Function (r = −0.77, p < 0.001), Role Function (r = −0.75, p < 0.001), Cognitive Function (r = −0.57, p < 0.001), Social Function (r = −0.69, p < 0.001) and positively with MY20 Disease Symptoms (r = 0.65, p < 0.001) and Side Effects of Treatment (r = 0.74, p < 0.001); MyPOS Emotional Response scores correlated negatively with EORTC-QLQ-C30 Emotional Function (r = −0.72, p < 0.001) and MY20 Future Perspectives (r = −0.77, P < 0.001); and MyPOS Healthcare Support scale did not correlate to r > 0.50 with any of the EORTC scales or single items.

## Discussion

This study reports the development of the MyPOS questionnaire with input from the literature, clinical staff and myeloma patients across all disease stages. The MyPOS is both brief and comprehensive, and pretesting has demonstrated good acceptability on cognitive assessment. The cross sectional survey of 380 patients showed a low time burden and few missing items, and found that the MyPOS is a reliable and valid tool with the ability to distinguish between clinically distinct groups, and good discriminant and divergent validity against subscales of the EORTC-QLQ-C30 and MY20.

### Cross sectional survey sampling

The use of consecutive enrolment for the psychometric evaluation ensured that the MyPOS was validated in a broadly clinically representative group. This is fitting for a questionnaire designed for clinical use, since validation should occur in a sample that reflects a questionnaire’s intended utility. The sample reflected the overall population of myeloma patients across all settings, including inpatients (4.7%), ECOG performance status 3–4 (9.5%), and those receiving high dose treatment with stem cell support (1.3%). The final sample of 380 participants was probably biased towards the more well patients, since 31 (6%) of the 517 patients screened were excluded or declined due to being too unwell, distressed or symptomatic. Further validation of the MyPOS specifically in the inpatient setting may be worthwhile, since this would probably capture more patients with poor performance status and receiving high dose treatment with stem cell support.

In contrast to the MyPOS, the EORTC-QLQ-C30 and its MY20 module were designed as research tools and much of their validation has (appropriately) taken place using myeloma patients recruited into clinical trials [9,11]. Such samples are much more highly selected and likely to be medically fitter and suffering from more acute treatment side effects as compared with clinically representative groups which will contain a mix of patients in the later stages of illness for whom different issues may be relevant to QOL. Research tools such as the EORTC questionnaires may not always, therefore, be well suited to clinical use [8] and this highlights the importance of tools like the MyPOS that have been developed specifically for use in clinical settings.

### MyPOS Symptoms & Function subscale

The exploratory factor analysis showed that MyPOS symptom items (e.g. pain; nausea; vomiting) loaded onto a single subscale with function items (e.g. usual activities; hobbies and leisure; time with family and friends). This aligns with the previously reported model of QOL in myeloma that showed the impact of physical symptoms on QOL is dependent on how much they affect activities, participation and emotional wellbeing [13]. The MyPOS asks respondents to consider the impact of symptoms on ‘activities or concentration’ whereas most other QOL questionnaires developed or used in myeloma ask only about the severity or frequency of symptoms and so many not capture all that is important to QOL [8].

### MyPOS Healthcare Support subscale

The MyPOS is the only myeloma-specific QOL questionnaire to contain a subscale dedicated to healthcare factors. Healthcare factors have been reported as important by myeloma patients in a number of qualitative studies [13,35-38], and they are useful for clinical teams at both an individual patient level (e.g. to highlight when a patient may require more information about their illness), and in aggregate (e.g. for auditing patient satisfaction across a service). This makes a strong case for such items to be included in myeloma QOL questionnaires, especially where they are designed with clinical use in mind. As research tools the EORTC-QLQ-C30 and FACT-MM contain no healthcare-related items. The EORTC myeloma-specific module initially included 4 such items as the MY24 [10] but these were subsequently removed due to ceiling effects and the module revised to the MY20 [9]. Interestingly, ceiling effects were also seen in 2 out of 5 items in the MyPOS Healthcare Support subscale, (Knowledge and skill of doctors and nurses; Care and respect of doctors and nurses), with no participant using the worst response options in each case. It was decided to leave these unused response options in the MyPOS, since their lack of use may represent a selection bias in favour of patients who are happy with their clinical team and so happy to participate in the validation study. It was noted that 28 (5%) of the 517 patients screened declined to participate but refused to give a reason why, raising the possibility that within this group are some patients who unwilling to participate as they were dissatisfied with their clinical care and might have used these unused response options.

The Healthcare Support subscale scores had a Cronbach’s α coefficient of 0.64 which was below the desired range of 0.7-0.9. This is probably due in part to the short length of this subscale (5 items). Raising the number of items in a scale can in itself raise the α coefficient, even when item correlations remain static [39,40]. High α coefficients can therefore be difficult to achieve in short scales such as the MyPOS Healthcare Support subscale.

### MyPOS diarrhoea item

This was the only MyPOS item without a factor loading of ≥0.30 on any subscale. This may be because the scores for this item were highly skewed, with 73% of respondents having no diarrhoea and less than 3% with severe or overwhelming diarrhoea. Severe or overwhelming diarrhoea is most likely in patients receiving high intensity hospital-based treatment such as autologous bone marrow transplantation, yet only 4.7% of participants in the cross sectional survey were inpatients. Whilst this low proportion of inpatients may be clinically representative, this resulted in problems such as overwhelming diarrhoea being less well represented the sample. The MyPOS steering group opted to retain the item on clinical grounds, since it was considered an important clinical problem and required for the MyPOS to have utility across different clinical settings.

### MyPOS sex item

The inclusion of an item about sex is an important strength of the MyPOS, since it is often omitted from QOL questionnaires for use in this group [8]. An earlier qualitative study of QOL in people with myeloma found that patients felt that sexual function was affected my myeloma and its treatment, but both patients and staff find sex difficult to broach in typical clinical consultation [13]. Examples of reported problems included vaginal dryness following chemotherapy, and concerns about sex whilst thrombocytopenic (impaired clotting of the blood) [13]. Including an item about sex items in the MyPOS may help empower patients to discuss hidden problems and allow the treating physician to offer appropriate advice, or trigger referral to other services [41,42].

In contrast to the MyPOS, the most widely used and validated existing QOL questionnaires in myeloma (the EORTC-QLQ-C30 and MY20) together contain no item about sex, although this was considered during the MY20’s development and highlighted as an area for future research [10]. The more recently developed FACT-MM questionnaire does contain the item “*I am satisfied with my sex life*”, with a five point Likert scale of responses [12]. The term ‘*sex life’* has been reported as the most encompassing for different aspects of intimacy [43], and so the prototype MyPOS item was worded “*Have you felt satisfied with your sex life*?”. However, participants in the cognitive interviews had difficulty with the word satisfaction, reporting that they could only be satisfied with their sex life after having sex, making the question irrelevant if no sexual activity had taken place. This problem occurred despite the lead-in statement: “*Please answer this question regardless of your current amount of sexual activity.*” The wording was therefore amended to “*Have you been worrying about your sex life*?” It is acknowledged that goes beyond rephrasing and changes the meaning of the question. However, the intended clinical utility of this item is to flag hidden problems that are difficult for patients and clinicians to raise. Asking about worries will still achieve this end, and was more acceptable to participants in the second round of cognitive interviews. The response rate in the cross sectional survey to the reworded MyPOS sex item was good for question of this kind, with only 8.4% missing data.

### Methodological limitations

An important limitation of the cognitive interviewing approach is the reliance on verbal report of cognitive processes that some people may not be able to articulate [20]. A larger sample size may also have yielded more refinements of the prototype MyPOS.

The use of consecutive enrolment to the cross sectional survey can be seen as both a strength and a weakness: the strength being the more clinically representative sample as compared with many other validation studies in myeloma [8]; the weakness being that the approach will not have accessed all available patients, for example those who were unable to attend a clinic appointment due to feeling unwell at home. The collection of demographic and clinical details from non-participants was incomplete due to ethical and data protection issues, meaning only limited comparisons could be made with the study sample. Bias in questionnaire responses may also have resulted from the 26.5% of participants who had help completing the questionnaire.

The use of a cross sectional design for the psychometric validation precluded evaluation of test-retest reliability and responsiveness to change, which require longitudinal data. True criterion validity could not be assessed in the absence of an accepted ‘gold standard’ measure of QOL in this group, so discriminant and divergent validity were assessed against the EORTC tools as an alternative.

## Conclusions

The MyPOS is the first myeloma-specific QOL questionnaire designed specifically for use in the clinical setting. The MyPOS was developed based on qualitative enquiry with patients and staff and has been refined with cognitive assessment. It is a brief, comprehensive and acceptable tool that is reliable and valid on psychometric testing. The MyPOS can now be used to support clinical decision making in the routine care of myeloma patients, although further validation in the inpatient setting may be of value, alongside longitudinal testing and evaluation with item response theory methods.

## Additional file

Additional file 1: Table S1. Probes used in cognitive interviews; **Table S2.** Prototype MyPOS before cognitive interviewing; **Table S3.** Sample characteristics for cognitive interviews; **Table S4.** Changes to MyPOS items and response options after cognitive interviews; **Table S5.** Revised MyPOS after cognitive interviews; **Table S6.** Comparison of participants and non-participants in the cross sectional survey.

## References

[1] GLOBOCAN 2012 v1.0, Cancer Incidence and Mortality Worldwide. IARC CancerBase No. 11 [Internet] [http://globocan.iarc.fr]

[2] Parkin M, Bray F, Ferlay J, Pisani P (2005). Global cancer statistics, 2002. CA Cancer J Clin.

[3] Gulbrandsen N, Wisloff F, Brinch L, Carlson K, Dahl IM, Gimsing P (2001). Health-related quality of life in multiple myeloma patients receiving high-dose chemotherapy with autologous blood stem-cell support. Med Oncol.

[4] Sherman AC (2004). Psychosocial adjustment and quality of life among multiple myeloma patients undergoing evaluation for autologous stem cell transplantation. Bone Marrow Transplant.

[5] Uyl-de Groot CA, Buijt I, Gloudemans IJM, Ossenkoppele GJ, Berg HP, Huijgens PC (2005). Health related quality of life in patients with multiple myeloma undergoing a double transplantation. Eur J Haematol.

[6] Sirohi B, Powles R (2006). Epidemiology and outcomes research for MGUS, myeloma and amyloidosis. Eur J Cancer.

[7] Sherman AC, Simonton S, Latif U, Plante TG, Anaissie EJ (2009). Changes in quality-of-life and psychosocial adjustment among multiple myeloma patients treated with high-dose melphalan and autologous stem cell transplantation. Biol Blood Marrow Transplant.

[8] Osborne TR, Ramsenthaler C, Siegert RJ, Edmonds PM, Schey SA, Higginson IJ (2012). What issues matter most to people with multiple myeloma and how well are we measuring them? A systematic review of quality of life tools. Eur J Haematol.

[9] Cocks K, Cohen D, Wisloff F, Sezer O, Lee S, Hippe E (2007). An international field study of the reliability and validity of a disease-specific questionnaire module (the QLQ-MY20) in assessing the quality of life of patients with multiple myeloma. Eur J Cancer.

[10] Stead ML, Brown JM, Velikova G, Kaasa S, Wisloff F, Child JA (1999). Development of an EORTC questionnaire module to be used in health-related quality-of-life assessment for patients with multiple myeloma. European Organization for Research and Treatment of Cancer Study Group on Quality of Life. Br J Haematol.

[11] Wisloff F, Eika S, Hippe E, Hjorth M, Holmberg E, Kaasa S (1996). Measurement of health-related quality of life in multiple myeloma. Nordic Myeloma Study Group. Br J Haematol.

[12] Wagner LI, Robinson DJ, Weiss M, Katz M, Greipp P, Fonseca R (2012). Content development for the Functional Assessment of Cancer Therapy-Multiple Myeloma (FACT-MM): use of qualitative and quantitative methods for scale construction. J Pain Symptom Manage.

[13] Osborne TR, Ramsenthaler C, de Wolf-Linder S, Schey SA, Siegert RJ, Edmonds PM (2014). What matters most to people with multiple myeloma? A qualitative study of views on quality of life. BMC Cancer.

[14] Palliative Care Outcome Scale. [http://www.pos-pal.org]

[15] Kvam AK, Fayers P, Wisloff F (2010). What changes in health-related quality of life matter to multiple myeloma patients? A prospective study. Eur J Haematol.

[16] Beatty PC, Willis GB (2007). Research synthesis: the practice of cognitive interviewing. Public Opin Quart.

[17] Brod M, Tesler LE, Christensen TL (2009). Qualitative research and content validity: developing best practices based on science and experience. Qual Life Res.

[18] Willis GB (2005). Cognitive interviewing in practice: think-aloud, verbal probing and other techniques. Cognitive interviewing: a tool for improving questionnaire design.

[19] Tourangeau R, Jabine TB, Straf ML, Tanur JM, Tourangeau R (1984). Cognitive sciences and survey methods. Cognitive aspects of survey methodology: building a bridge between disciplines.

[20] Collins D (2003). Pretesting survey instruments: an overview of cognitive methods. Qual Life Res.

[21] Willis GB (2005). Analyzing and documenting cognitive interview results. Cognitive interviewing: a tool for improving questionnaire design.

[22] Nunally JO (1978). Psychometric theory.

[23] Guilford JP (1956). Psychometric methods.

[24] Tabachnick BG, Fidell LS, Tabachnick BG, Fidell LS (2012). Chapter 13: principal components and factor analysis. Using multivariate statistics.

[25] Bartlett MS (1950). Tests of significance in factor analysis. Br J Psychol.

[26] Kaiser HF (1970). A second-generation little jiffy. Psychometrika.

[27] Kaiser HF (1974). Little Jiffy, Mark IV. Educ Psychol Meas.

[28] Cattell RB (1966). The scree test for the number of factors. Multivar Behav Res.

[29] O’Connor BP (2000). SPSS and SAS programs for determining the number of components using parallel analysis and Velicer’s MAP test. Behav Res Methods Instrum Comput.

[30] Velicer WF, Jackson DN (1990). Component analysis versus common factor-analysis - Some further observations. Multivar Behav Res.

[31] Fayers PM, Machin D (2007). Scores and measurements: vaidity, reliability, sensitivity. Quality of life: the assessment, analysis and interpretation of patient reported outcomes.

[32] Johnson C, Aaronson N, Blazeby JM, Bottomley A, Fayers P, EORTC Quality of Life Group, et al. Guidelines for developing questionnaire modules (4th Edition). Available online at http://groupseortcbe/qol/sites/default/files/archives/guidelines_for_developing_questionnaire-_final.pdf 2011.

[33] de Vet HCW, Terwee CB, Mokkink LB, Knol DL (2011). Field testing: item reduction and data structure. Measurement in medicine.

[34] Fayers P, Aaronson NK, Bjordal K, Curran D, and Groenvold M on behalf of the EORTC Quality of Life Study Group (2001). EORTC QLQ-C30 Scoring Manual (Third edition).

[35] Dahan J, Auerbach CF (2006). A qualitative study of the trauma and posttraumatic growth of multiple myeloma patients treated with peripheral blood stem cell transplant. Pall Supp Care.

[36] Maher K, De Vries K (2011). An exploration of the lived experiences of individuals with relapsed Multiple Myeloma. Eur J Cancer Care.

[37] Molassiotis A, Wilson B, Blair S, Howe T, Cavet J (2011). Living with multiple myeloma: experiences of patients and their informal caregivers. Supp Care Cancer.

[38] Potrata B, Cavet J, Blair S, Howe T, Molassiotis A (2011). Understanding distress and distressing experiences in patients living with multiple myeloma: an exploratory study. Psycho-Oncol.

[39] Cortina JM (1993). What is coefficient alpha? An examination of theory and applications. J Appl Psychol.

[40] Streiner DL (2003). Starting at the beginning: an introduction to coefficient alpha and internal consistency. J Pers Assess.

[41] Higginson IJ, Carr A (2001). Using quality of life measures in the clinical setting. BMJ.

[42] Osoba D (2007). Translating the science of patient-reported outcomes assessment into clinical practice. J Natl Cancer Inst Monogr.

[43] Fortune-Greeley AK, Flynn KE, Jeffery DD, Williams MS, Keefe FJ, Reeve BB (2009). Using cognitive interviews to evaluate items for measuring sexual functioning across cancer populations: improvements and remaining challenges. Qual Life Res.

