# Supplementary material for: Improving the assessment of quality of life in the clinical care of myeloma patients: the development and validation of the Myeloma Patient Outcome Scale (MyPOS)
Source: BMC Cancer. 2015 Apr 14;15:280. doi: 10.1186/s12885-015-1261-6 (PMC4404617; doi:10.1186/s12885-015-1261-6)
Supplement: Additional file 1: Table S1. — Probes used in cognitive interviews; Table S2. Prototype MyPOS before cognitive interviewing; Table S3. Sample characteristics for cognitive interviews; Table S4. Changes to MyPOS items and response options after cognitive interviews; Table S5. Revised MyPOS after cognitive interviews; Table S6. Comparison of participants and non-participants in the cross sectional survey. [file 12885_2015_1261_MOESM1_ESM.pdf]

Table S1: Probes used in cognitive interviews, adapted from [20]

**General:**	• What were you thinking about when you answered that question?<br>• I noticed you hesitated before answering that question – what were you thinking about?<br>• Is there anything else you would like to say about this question?<br>• Is there anything else you would like to say about the questionnaire as a whole?
**Comprehension:**<br><br>*What does the respondent believe the question to be asking?*	• What does the question mean to you, in your own words?<br>• What does the word XXX mean to you?<br>• How easy or difficult was it to understand this question?<br>• How would you change this question to make it easier to understand?
**Retrieval:**<br><br>*Can the respondent recall the information required by the question? Is the recall period suitable?*	• How well could you remember your experience when answering this question?<br>• Was it easy or difficult to think about the past week when answering this question?<br>• Would there be a different time period that would be easier to understand?
**Judgement:**<br><br>*Is the respondent able to make an evaluation based on the information recalled?*	• What were you thinking about when you answered this question?<br>• How did you arrive at your answer to that question?<br>• Was that easy or hard to arrive at your answer? Why do you say that?<br>• How sure are you of the answer to this question?
**Response:**<br><br>*Is the respondent able to map their internally generated answer to a response option?*	• How did you choose your answer to this question?<br>• Was it hard or easy to select an answer from the options given?<br>• Did all options make sense for this question?
**Acceptability:**<br><br>*Is the question acceptable to respondents?*	• Were there any other issues with this question?<br>• Did you find this question upsetting?<br>• Did you find this question embarrassing?<br>• Did you find this question inappropriate?

Table S2: Prototype MyPOS before cognitive interviewing

**1.**	If any, what have been your main problems <u>over the past week</u>?	[Open question with three empty boxes for respondent to complete, numbered 1-3]				
**2.**	Below is a list of symptoms, which you may or may not have experienced. Please put a tick in the box to show how each of these symptoms <u>has affected you over the past week:</u>	**Not at all**<br>No effect	**Slightly**<br>But not bothered to be rid of it	**Moderately**<br>Limits some activity or concentration	**Severely**<br>Activities or concentration markedly affected	**Overwhelmingly**<br>Unable to think of anything else
**A**	Pain					
**B**	Fatigue or lack of energy					
**C**	Shortness of breath					
**D**	Diarrhoea					
**E**	Constipation					
**F**	Nausea (feeling like you are going to be sick)					
**G**	Vomiting (being sick)					
**H**	Mouth problems					
**I**	Poor mobility					
**J**	Tingling in the hands and feet					
**K**	Difficulty remembering things					
**L**	Please also describe the effects of any other symptoms you may have: [Three boxes for respondent to add additional symptoms, numbered 1-3]					
**3.**	Over the past week, have you been able to carry out your usual daily activities without help from others?	**Yes, as much as I wanted**	**Most of the time**	**Sometimes**	**Occasionally**	**No, not at all**
**4.**	Over the past week, have you been able to take part in social activities?					
**5.**	Over the past week, have you been able to pursue your hobbies and leisure activities?					
**6.**	Over the past week, have you been able to spend quality time with family and friends?					
**7.**	*Please answer this question regardless of your current amount of sexual activity. Or tick here to skip this question:* ❑<br><br>Over the past week, have you felt satisfied with your sex life?	**Yes, always**	**Most of the time**	**Sometimes**	**Occasionally**	**No, not at all**
**8.**	Over the past week, have you been feeling depressed?	**No, not at all**	**Occasionally**	**Sometimes**	**Most of the time**	**Yes, always**
**9.**	Over the past week, have you been feeling anxious or worried about your illness or treatment?					
**10.**	Over the past week, have you been worrying about getting infections?					
**11.**	Over the past week, have you been worrying about your physical appearance?					
**12.**	Over the past week, have you been worrying about your financial situation?					
**13.**	Over the past week, have you been worrying that your illness will get worse?					
**14.**	Over the past week, have you been worrying about dying?					
**15.**	Over the past week, have you felt able to cope with your illness and treatment?	**Yes, always**	**Most of the time**	**Sometimes**	**Occasionally**	**No, not at all**
**16.**	Over the past week, have you felt satisfied with the service your doctors and nurses provide?					
**17.**	Over the past week, has it been possible to contact your doctors or nurses for advice if needed?					
**18.**	Over the past week, have you felt adequately informed about your illness?	**Full Information**<br>or as much as I wanted	**Information received**<br>but hard to understand	**Information received**<br>but would have liked more	**Very little information**<br>and some questions were avoided	**Not at all**<br>And wanted information
**19.**	Over the past week, have you felt adequately informed about your treatment?					
**20.**	Over the past week, have you felt adequately informed about what might happen in the future?					
**21.**	How did you complete this questionnaire?	**On my own**	**With the help of a friend or relative**	**With help from a staff member**		

Wording of items 1, 2, A, C, E, F, G, H, 8, 9 and 21 and all response options were taken from the Palliative Care Outcome Scale (POS) or its accompanying symptom scale the POS-Symptoms [14]

Table S3: Sample characteristics for cognitive interviews (n = 12)

**Setting of interview**	
Hospital outpatient	3
Hospital inpatient	3
Participant's home	6
**Gender**	
Male	6
Female	6
**Age**	
Median (range)	64.5 (51-76)
<65	6
≥65	6
**Marital status**	
Single	2
Married / partnered	9
Widowed	1
**Ethnicity**	
White British	8
White Other	1
Black	3
**Religion**	
Atheist	2
Christian	9
Other	1
**Highest educational level**	
Did not finish school	2
Secondary school graduate	4
College / technical qualification	5
University first degree	1
**Occupation status**	
Working or student	3
Not working	3
Retired	6
**ECOG performance status**	
0-2	7
3-4	5
**Treatment status**	
On treatment	6
Off treatment	6
**Disease phase**	
Newly diagnosed	3
Stable / plateau phase	4
Relapsed / progressive	5
**Immunoglobulin type**	
IgG	4
IgA	2
IgM	1
Light chain	5
**ISS Stage at diagnosis**	
II	2
III	4
Not known	6
**Months since diagnosis**	
0-12	3
13-24	1
25-36	2
37-48	2
Over 48	4

Table S4: Changes to MyPOS items and response options after cognitive interviews

Prototype MyPOS items / responses	Problems identified with supporting quotations	Revised MyPOS items / responses
If any, what have been your main problems <u>over the past week</u>?<br><br>*[Open question with 3 lines beneath]*	Some clinical problems had arisen and gone away within the past week and so were no longer relevant.	What are your main problems or concerns at the moment?
Below is a list of symptoms, which you may or may not have experienced. Please put a tick in the box to show how each of these symptoms <u>has affected you over the past week:</u><br><br>**Not at all** – No effect<br>**Slightly** – But not bothered to be rid of it<br>**Moderately** – Limits some activity or concentration<br>**Severely** – Activities or concentration markedly affected<br>**Overwhelmingly** – Unable to think of anything else	Unclear instructions leading to more than one response:<br><br>*"It doesn't say whether I can give more than one answer. I will tick moderately and put 70% and tick severely and put 30%"*<br><br>Response options hard to understand. Respondents required to consider the effect of each symptom on activities or concentration AND whether they wanted rid of it. No suitable response if the symptom was affecting them 'Slightly' and they still wanted rid of it:<br><br>*"Limits some activities or concentration, you could use that I think … It's this one 'not bothered to be rid of it' [that I'm having trouble with]"*	Below is a list of symptoms, which you may or may not have experienced. For each symptom please <u>tick one box</u> that best describes how it has affected you <u>over the past week</u>:<br><br>**Not at all** – I have not had this symptom in the past week<br>**Slightly** – Little or no effect on activities or concentration<br>**Moderately** – Some effect on activities or concentration<br>**Severely** – Marked effect on activities or concentration<br>**Overwhelmingly** – Unable to think of anything else
Tingling in the hands and feet	Question did not apply if tingling was only present in the hands OR the feet.	Tingling in the hands and / or feet
Please also describe the effects of any other symptoms you may have:<br><br>*[Open question but with the same structured response options as for other symptom items]*	Unclear if asking for *additional* symptoms not listed in the structured items:<br><br>*"I'm not quite sure whether I'm just repeating … I'm not sure what it's asking me to do there"*<br><br>Unclear recall period:<br><br>*"Well you haven't actually said 'over the past week'"*	Please list any <u>other</u> symptoms not mentioned above, and tick <u>one box</u> to show how they have affected you <u>over the past week</u>:
Over the past week, have you been able to carry out your usual daily activities without help from others?	'Usual daily activities' taken to mean only those activities done every day – thereby excluding activities such as grocery shopping or attending place of work Mon-Fri.	Over the past week, have you been able to carry out your usual activities without help from others?
Over the past week, have you been able to take part in social activities?	Meaning of 'social activities' unclear:<br><br>*"I wonder what the meaning of social activities … do they mean a club or association? … going to the shop? … being at work? It's not entirely clear"*<br><br>Conceptual overlap with items about 'hobbies and leisure activities' and 'quality time with family and friends':<br><br>*"Same as [the item about hobbies and leisure activities] really. It's the same thing isn't it virtually"*<br>*"I've answered that one there … my social activity this week has been seeing family and friends"*	Item removed.<br><br>Concept is confusing to respondents and covered by other items.
Please answer this question regardless of your current amount of sexual activity. Or tick here to skip this question: ❑<br>Over the past week, have you felt satisfied with your sex life?	Meaning of *satisfaction* with sex life unclear. Satisfaction seen as something that can only occur after sex, rather than being satisfied even in the absence of sex:<br><br>*"I think it's saying … am I able to have sex"*<br>*"It means, first of all, have I had sex … and if I have had sex was I satisfied? Was it good sex, you know"*	<u>We would like you to answer this question whether or not you are sexually active.</u> *Or if you prefer not to answer then please tick here:* ❑<br>Over the past week, have you been worrying about your sex life?
Over the past week, have you been worrying about getting infections?	Question may not apply to those who currently have an infection.	Over the past week, have you been worrying about infections?

Prototype MyPOS items / responses	Problems identified with supporting quotations	Revised MyPOS items / responses
Over the past week, have you been worrying about dying?	Distressing item, particularly if newly diagnosed:<br><br>*"I personally wouldn't … put it in. You ask me a question like that probably I'd start crying"*<br>*"That could be very concerning"*<br>*"It's an extremely difficult question"*<br><br>Overlap between 'worrying about dying' and 'worrying that your illness will get worse':<br><br>*"Those two are linked to me"*<br>*"It's over to the next question isn't it really"*	Item removed<br><br>May cause distress to respondents and concept is covered by other items.
Over the past week, has it been possible to contact your doctors or nurses for advice if needed?<br><br>Over the past week, have you felt satisfied with the service your doctors and nurses provide?	Recall period of 'the past week' confusing if no contact with healthcare providers in that time:<br><br>*"Because I haven't seen them … so the last week wouldn't apply"*<br><br>Different interpretations of 'satisfied with the service' with no consensus amongst respondents.	Are you able to contact your doctors or nurses for advice if needed?<br><br>Do your doctors and nurses show a good standard of knowledge and skill when treating you?<br><br>Do your doctors and nurses show care and respect when treating you?
Over the past week, have you felt adequately informed about your illness?<br><br>Over the past week, have you felt adequately informed about your treatment?<br><br>Over the past week, have you felt adequately informed about what might happen in the future?<br><br>**Full information** – or as much as I wanted<br>**Information received** – but hard to understand<br>**Information received** – but would have liked more<br>**Very little information** – and some questions were avoided<br>**Not at all** – and wanted information	Recall period of 'the past week' confusing:<br><br>*"I can say yes, but no-one has spoken to me over the last week about it"*<br>*"It would be 'no information' because nobody has spoken to me this week … the last time I spoke to anybody was possibly three weeks ago"*<br>*"It wasn't easy thinking about the past week … this question really is beyond the past week"*<br><br>Separation of 'illness' and 'treatment' into two items illogical:<br><br>*"To me [illness and treatment are] one in the same. I would put them in the same question, because they go hand-in-hand"*	Do you have enough information about your illness and treatment?<br><br>Do you have enough information about what might happen to you in the future?<br><br>**Enough information** – the right amount for me<br>**Information received** – but hard to understand<br>**Information received** – but would like more<br>**Very little information** – and would like more<br>**Not at all** – and would like information

Table S5: Revised MyPOS after cognitive interviews

**1.**	[a]What are your main problems or concerns at the moment?	[Open question with three empty boxes for respondent to complete, numbered 1-3]				
**2.**	[a]Below is a list of symptoms, which you may or may not have experienced. For each symptom please <u>tick one box</u> that best describes how it has affected you <u>over the past week</u>:	**Not at all**<br>[a]I have not had this symptom in the past week	**Slightly**<br>[a]Little or no effect on activities or concentration	**Moderately**<br>[a]Some effect on activities or concentration	**Severely**<br>[a]Marked effect on activities or concentration	**Overwhelmingly**<br>Unable to think of anything else
**A.**	Pain					
**B.**	Fatigue or lack of energy					
**C.**	Shortness of breath					
**D.**	Diarrhoea					
**E.**	Constipation					
**F.**	Nausea (feeling like you are going to be sick)					
**G.**	Vomiting (being sick)					
**H.**	Mouth problems					
**I.**	Poor mobility					
**J.**	[a]Tingling in the hands and / or feet					
**K.**	Difficulty remembering things					
**L.**	[a]Please list any <u>other</u> symptoms not mentioned above, and tick <u>one box</u> to show how they have affected you <u>over the past week</u>: [Three boxes for respondent to add additional symptoms, numbered 1-3]					
**3.**	[a]Over the past week, have you been able to carry out your usual activities without help from others?	**Yes, as much as I wanted**	**Most of the time**	**Sometimes**	**Occasionally**	**No, not at all**
**4.**	Over the past week, have you been able to pursue your hobbies and leisure activities?					
**5.**	Over the past week, have you been able to spend quality time with family and friends?					
**6.**	[a]<u>We would like you to answer this question whether or not you are sexually active.</u> *Or if you would prefer not to answer then please tick here:* ❑<br>[a]Over the past week, have you been worrying about your sex life?	**[a]No, not at all**	**[a]Occasionally**	**[a]Sometimes**	**[a]Most of the time**	**[a]Yes, always**
**7.**	Over the past week, have you been feeling depressed?	**No, not at all**	**Occasionally**	**Sometimes**	**Most of the time**	**Yes, always**
**8.**	Over the past week, have you been feeling anxious or worried about your illness or treatment?					
**9.**	[a]Over the past week, have you been worrying about infections?					
**10.**	Over the past week, have you been worrying about your physical appearance?					
**11.**	Over the past week, have you been worrying about your financial situation?					
**12.**	Over the past week, have you been worrying that your illness will get worse?					
**13.**	Over the past week, have you felt able to cope with your illness and treatment?	**Yes, always**	**Most of the time**	**Sometimes**	**Occasionally**	**No, not at all**
**14.**	[a]Are you able to contact your doctors or nurses for advice if needed?					
**15.**	[a]Do your doctors and nurses show a good standard of knowledge and skill when treating you?					
**16.**	[a]Do your doctors and nurses show care and respect when treating you?					
**17.**	[a]Do you have enough information about your illness and treatment?	**[a]Enough Information**<br>[a]the right amount for me	**Information received**<br>but hard to understand	**[a]Information received**<br>[a]but would like more	**[a]Very little information**<br>[a]and would like more	**[a]No information received**<br>[a]and would like information
**18.**	[a]Do you have enough information about what might happen to you in the future?					
**19.**	How did you complete this questionnaire?	**On my own**	**[a]With help from a friend or relative**	**With help from a staff member**		

[a]Wording changed following cognitive interviews

Table S6: Comparison of participants and non-participants in the cross sectional survey

	Participants (n = 380)	Non-Participants (n = 137)
**Gender**		
Male	231 (60.8%)	58 (42.3%)
Female	149 (39.2%)	42 (30.7%)
Not known	0	37 (27%)
**Age**		
<65	113 (29.7%)	34 (24.8%)
≥65	267 (70.3%)	64 (46.7%)
Not known	0	39 (28.5%)
**Treatment status**		
On treatment	181 (47.6%)	40 (29.2%)
Off treatment	199 (52.4%)	35 (25.5%)
Not known	0	62 (45.3%)
**Disease phase**		
Newly diagnosed	79 (20.8%)	15 (10.9%)
Stable / plateau phase	174 (45.8%)	43 (31.4%)
Relapsed / progressive	127 (33.4%)	27 (19.7%)
Not known	0	52 (38.0%)
